# Formononetin alleviates acute pancreatitis by reducing oxidative stress and modulating intestinal barrier

**DOI:** 10.1186/s13020-023-00773-1

**Published:** 2023-06-27

**Authors:** Jun Yang, Xiaowei Sha, Di Wu, Bo Wu, Xiaohua Pan, Li-Long Pan, Yuanlong Gu, Xiaoliang Dong

**Affiliations:** 1 Wuxi School of Medicine, Jiangnan University, Wuxi, Jiangsu People’s Republic of China; 2 Affiliated Hospital of Jiangnan University, Wuxi, Jiangsu People’s Republic of China; 3 School of Food Science and Technology, Jiangnan University, Wuxi, Jiangsu People’s Republic of China

**Keywords:** Formononetin, Acute pancreatitis, Intestinal injury, Nuclear factor erythroid2-related factor 2, Kelch like ECH Associated protein 1, Reactive oxygen species

## Abstract

**Background:**

Acute pancreatitis (AP) is a recurrent inflammatory disease. Studies have shown that intestinal homeostasis is essential for the treatment of AP. Formononetin is a plant-derived isoflavone with antioxidant properties that can effectively treat a variety of inflammatory diseases. This study aims to investigate the role of formononetin in protecting against AP and underlying mechanism.

**Methods:**

Caerulein was used to induce AP. The inflammatory cytokines were detected using Quantitative real-time PCR and commercial kits. Histological examination was applied with hematoxylin and eosin staining. Western blot was conducted to detect expression of intestinal barrier protein and signaling molecular. Molecular docking was performed to assess protein-ligand interaction.

**Results:**

In this study, we found formononetin administration significantly reduced pancreatic edema, the activities of serum amylase, lipase, myeloperoxidase, and serum endotoxin. The mRNA levels of inflammatory cytokines such as tumor necrosis factor α, monocyte chemoattractant protein-1, interleukin-6, and interleukin-1 beta (IL-1β) in pancreas were also significantly decreased by formononetin. The following data showed formononetin pretreatment up-regulated the expressions of tight junction proteins in the colon, and decreased *Escherichia coli* translocation in the pancreas. In addition, formononetin inhibited the activation of nucleotide-binding oligomerization domain leucine-rich repeat and pyrin domain-containing 3 in pancreatic and colonic tissues of AP mice. Moreover, formononetin activated Kelch Like ECH Associated Protein 1 (Keap1) / Nuclear factor erythroid2-related factor 2 (Nrf2) signaling pathway to reduce reactive oxygen species (ROS) levels. Docking results showed that formononetin interact with Keap1 through hydrogen bond.

**Conclusions:**

These findings demonstrate that formononetin administration significantly mitigate AP through reducing oxidative stress and restoring intestinal homeostasis, and provide insights into the new treatment for AP.

**Supplementary Information:**

The online version contains supplementary material available at 10.1186/s13020-023-00773-1.

## Introduction

Acute pancreatitis (AP) is a recurrent inflammatory disease with a high incidence, characterized by a sudden onset and rapid disease progression [1, 2]. It has been reported that approximately 20% of patients develop moderate or severe AP with pancreatic or peripancreatic tissue necrosis or organ failure, or both, with a mortality rate of 20–40% [3, 4]. However, the treatment of pancreatitis is still an unsolved problem. It is particularly urgent to explore a new strategy to suppress the AP inflammatory response.

During AP, the massive release of inflammatory factors such as tumor necrosis factor α (TNF-α) cause intestinal mucosa damage, and these factors can further promote the activation of inflammatory mitogen-activated protein kinase (MAPK) and nucleotide-binding oligomerization domain leucine-rich repeat and pyrin domain-containing 3 (NLRP3) pathways and the release of inflammatory mediators, leading to more severe intestinal inflammation [5–7]. In addition, AP induced oxidative stress can further amplify inflammatory response, and excessive production of reactive oxygen species (ROS) may also directly destroy intestinal barrier function [8]. Accompanied with intestinal inflammation and oxidative stress, the intestinal mucosal barrier function is impaired, characterized by downregulating expression of epithelial tight junction proteins (TJPs), which leads to migration of bacteria and toxins into blood or pancreas and results in secondary pancreatic and peripancreatic infection [8, 9]. Therefore, enhancing intestinal barrier function and inhibiting oxidative stress are promising for the prevention and treatment of AP.

Plenty of edible natural products have the potential to exert anti-inflammatory and anti-oxidative effects. Formononetin is an isoflavone isolated from herbal as well as edible plants and is considered to have many pharmacological properties such as anticancer [10], anti-inflammatory [11] and antioxidant properties [12]. Earlier studies have demonstrated that formononetin had a protective effect on dextran sulfate-induced acute colitis by inhibiting NLRP3 inflammatory vesicle pathway [13]. Research shows that formononetin treatment can reshape the gut microflora, and improve metabolic complications and systemic inflammation via modulation of the gut bacteria in mice. Formononetin can affect certain bacterial phyla and reshape the gut microbiota, and maintains the integrity of the intestinal membrane by regulating the expression of Muc-2 and occluding [14]. All these lead us to wonder whether formononetin has a protective role in AP. In this study, we demonstrated the beneficial effects of formononetin on AP and associated intestinal injury, and mechanistically formononetin reduces ROS-mediated inflammation response through Kelch Like ECH Associated Protein 1 (Keap1)- Nuclear factor erythroid2-related factor 2 (Nrf2) signaling pathway, ultimately inhibits intestinal barrier dysfunction and bacterial translocation to alleviate AP.

## Materials and methods

### Animals

Male C57BL/6J mice (seven-week-old) were purchased from Charles River (Zhejiang, China) in this study. Mice were bred at the Animal Housing Unit of Jiangnan University (Wuxi, Jiangsu, China) under 23–25 ℃ and 12 h light-dark cycle with unlimited access to food and water. All mice were allowed to acclimatize to the laboratory conditions over the course of 1 week prior to the experiments. Animals were maintained in accordance with the guidelines of the National Institutes of Health. All experiments and protocols complied with the ARRIVE guidelines [15], and were approved by the animal ethics committee of Jiangnan University (JN. No20200430c0401030[037]).

### Induction of AP and pretreatment with formononetin

All mice were adjusted to laboratory conditions over the course of 1 week prior to the experiments and fasted for 12 h before induction of AP. Mice (20 ± 2 g) were randomly divided into three groups (n ≥ 6): control group, caerulein group and caerulein + formononetin group. Caerulein was purchased from Jiangsu Ji Tai Peptide Industry Science and Technology Co., Ltd. Formononetin (Cat. 485-72-3) was purchased from Shanghai Aladdin Bio-Chem Technology Co., Ltd. The formononetin is dissolved in 1% dimethyl sulfoxide (DMSO). Remain heated while the drug is dissolved. To examine the biological effects of formononetin, mice were treated with formononetin (25, 50, 100 mg/kg/day) by gavage once a day for 7 consecutive days before induction of AP. Control and caerulein groups were given DMSO (1%) by gavage every day for 7 consecutive days. The mice received hourly intraperitoneal injections with normal saline or saline containing caerulein (50 µg/kg) for 8 h to induce AP. One hour after the last caerulein injection, mice were sacrificed by a lethal dose of pentobarbitone. Plasma and pancreatic tissue samples were harvested for subsequent assays.

### Serum amylase and lipase activity

Harvested blood was centrifuged at 3000 × g for 15 min after coagulating at room temperature for 25 min. The serum was then collected and stored at −80 ℃ until analysis. Serum amylase and lipase activity were determined by serum amylase assay kit (Jian Cheng Bioengineering Institute, Nanjing, China) and serum lipase assay kit (Jian Cheng Bioengineering Institute, Nanjing, China) according to the protocol in the instruction manual.

### Determination of pancreatic edema

The edema of the pancreas was quantified by the ratio of wet weight to dry weight. A portion of freshly harvested pancreas was defined as wet weight. The weight of the same sample after desiccation at 60 ℃ for 72 h was served as dry weight.

### Determination of myeloperoxidase (MPO) activity in serum

MPO activity was measured by using MPO assay kit (A044-1-1, Jian Cheng Bioengineering Institute, Nanjing, China) according to the protocol. Briefly, animal tissue samples were prepared into tissue homogenate with normal saline by weight volume ratio, and supernatant was taken by centrifugation for determination. Add the sample and reagents R2 and R3 as per the procedure, and bathe at 37  ℃ for 15 min. The above mixture was added with R4 and color developing agent and bathed at 37℃ for 30 min. Reagent R7 was added into the water bath at 60 ℃ for 10 min, and colorimetry was carried out at 460 nm wavelength and 1 cm optical diameter.

### Determination of lipopolysaccharide (LPS) in serum

An appropriate amount of serum was taken to measure LPS activity according to the instructions of LPS ELISA kit (Enzyme-linked Biotechnology, Shanghai, China). The kit was removed from the refrigerated environment, balanced at room temperature for 20 min, then the sample and biotin labeled antibody were added and incubated in a 37 ℃ water bath for 60 min. Remove the liquid and add the washing solution for 5 times. Substances were added and incubated at 37 ℃ for 15 min without light. Finally, absorbance was measured at 450 nm by a microplate reader Multiclan GO (Thermos Fisher Scientific Inc, Vantaa, Finland).

### Quantitative real-time PCR (qRT-PCR)

Total RNA was extracted from tissues using RNAiso Plus (TaKaRa, 9109, Japan) following the manufacturer’s protocol and cDNAs were synthesized by a reverse transcription reagent kit (TaKaRa, RR036A, Japan). Quantitative PCR analysis was performed using the Bio-Rad SYBR Green Supermix dye and specific primers for the gene in the Bio-Rad CFX Connect Real-Time System (CA, USA). The primers used in this study are provided in Additional file 1: Table S1. β-actin was used as a housekeeping gene.

### Histological examination

Freshly harvested pancreatic and colonic samples were fixed with 4% paraformaldehyde, dehydrated in ethanol, and then embedded with paraffin. Prepared sections were diced 5 μm sections by the Skiving Machine Slicer (Leica RM2245, Wetzlar, Germany) and then stained with hematoxylin and eosin (H&E) following the standard procedure. Morphological changes of pancreas and colon were examined under a Digital slice scanner (Pannoramic MIDI, 3DHISTCH, Hungary) at 200 × magnification (Additional file 2: Fig S1) The pancreatic pathological scoring analysis was performed according to the severity and extent of edema, necrosis, hemorrhage and inflammation as described by Schmidt [16].

### Co-immunoprecipitation and immunoblot analysis

Pancreatic and colonic tissues were homogenized in RIPA buffer (Thermo Fisher Scientific, MA, USA) with protease and phosphatase inhibitor cocktails (Sigma-Aldrich, MO, USA). Protein concentrations were determined by using a BCA protein assay kit (Beyotime, Shanghai, China). For Co-immunoprecipitation, proteins were immunoprecipitated with anti-keap1 antibody (10503-2-AP, Proteintech, Wuhan, China). The precleared protein A/G magnetic beads (HY-K0202, MedChemExpress, Shanghai, China) were incubated with immunocomplexes and washed with the lysis buffer. Protein samples after sodium dodecyl sulfate polyacrylamide gel electrophoresis (SDS-PAGE) were transferred on the nitrocellulose membrane (Millipore, MA, USA). The membranes were blocked with 5% w/v nonfat dry milk in TBS-T for 1 h at room temperature. In order to probe proteins with different molecular weights, the membranes were horizontally cut according to the markers and further incubated with appropriately diluted primary antibodies overnight at 4 ℃ and probed with secondary peroxidase-labeled antibody for 1 h at room temperature. Antibodies for Nrf2 (12,721 S), apoptosis-associated speck-like protein containing CARD (ASC) (67,824 S), Occludin (2847T) were purchased from Cell Signaling Technology (MA, USA). Cleaved-IL-1β (sc-23,460), Caspase-1-p20 (sc-398,715) were purchased from Santa Cruz Biotechnology (CA, USA). Antibodies for Keap1 (A1820), Heme oxygenase-1 (HO-1) (A19062) were purchased from ABclonal Technology Co., Ltd. Antibodies for Claudin-1 (ab180158), IL-18 (ab71495) were purchased from Abcam (Cambridge, UK). Antibodies for NLRP3 (ET1610-93), Zonula Occludens-2 (ZO-2) (40-2200) were purchased from HuaAn Biotechnology (Hangzhou, China) and Invitrogen (CA, USA) respectively. Blots were developed by enhanced chemiluminescence (Universal Hood III, Bio-Rad. USA).

### Measurement of pancreatic and colonal ROS generation

Fresh tissues from the pancreas and colon were embedded in optimal cutting temperature (OCT) compound (#4583; SAKURA, US), and then, the samples were cut into 7 mm sections. A working reagent of DHE (#S0063; Beyotime, Shanghai, CN) was dropped onto each section, and slides were incubated for 30 min at 37 ℃. Slides were placed in phosphate buffer saline (PBS) (pH = 7.4) and washed 3 times, each time for 5 min. Then, the tissues were incubated with 4′, 6-diamidino-2-phenylindole, dihydrochloride (DAPI, Wuhan Servicebio Technology Co., Ltd., Wuhan, China) solution at room temperature for 10 min and washed again. Finally, the slides were observed under a fluorescence microscope.

### Superoxide dismutase (SOD) detection

SOD activity was measured by using SOD assay kit (S0101S, Beyotime Biotechnology, Shanghai, China) according to the protocols provided by the supplier. Briefly, tissue samples were obtained from animals after blood removal by perfusion with saline (0.9% NaCl, containing 0.16 mg/ml heparin sodium). Appropriate tissue samples were homogenized at 4 ℃ or in an ice bath at the ratio of adding 100 µL of SOD sample preparation solution per 10 mg of tissue (glass homogenizers or various common electric homogenizers can be used). After centrifugation at 4 ℃ at about 12,000 g for 3–5 min, supernatant was taken as the sample to be tested. Add the sample to be tested and various other solutions in turn. Mix thoroughly after adding the reaction start-up solution. The calculation formula of SOD enzyme activity is as follows: SOD activity unit in the sample to be tested = SOD activity unit in the detection system = percentage of inhibition/(1- percentage of inhibition) units.

### Molecular docking study of formononetin

The chemical structure of formononetin was obtained from the PubChem database (Compound CID: 5,280,378), and converted to 3D structure by the Open Babel 2.4.1 software. The crystal structure of Keap1 (PDB ID: 5CGJ) was obtained from the RCSB protein database (www.rcsb.org). The receptor protein was removed ligand and water by the PyMOL 2.2.0 software. The docking process was performed with AutoDock Vina 1.1.2 software. From the docking results, the best conformation with the lowest docked energy was selected for further mapping analysis. Identification of non-covalent interactions between Keap1 and formononetin was conducted using Protein-Ligand Interaction Profiler (https://plip-tool.biotec.tu-dresden.de/plip-web/plip/index) [17].

### Statistical analysis

All data are expressed in terms of the mean ± SD. p < 0.05 was considered as a statistically significant difference. Statistical analyses were performed by one-way ANOVA analysis of variance followed by Dunnett’s test as a post hoc test using GraphPad Prism 8.0.1 (San Diego, USA).

## Results

### Formononetin treatment reduced the severity of AP

**Fig. 1:**
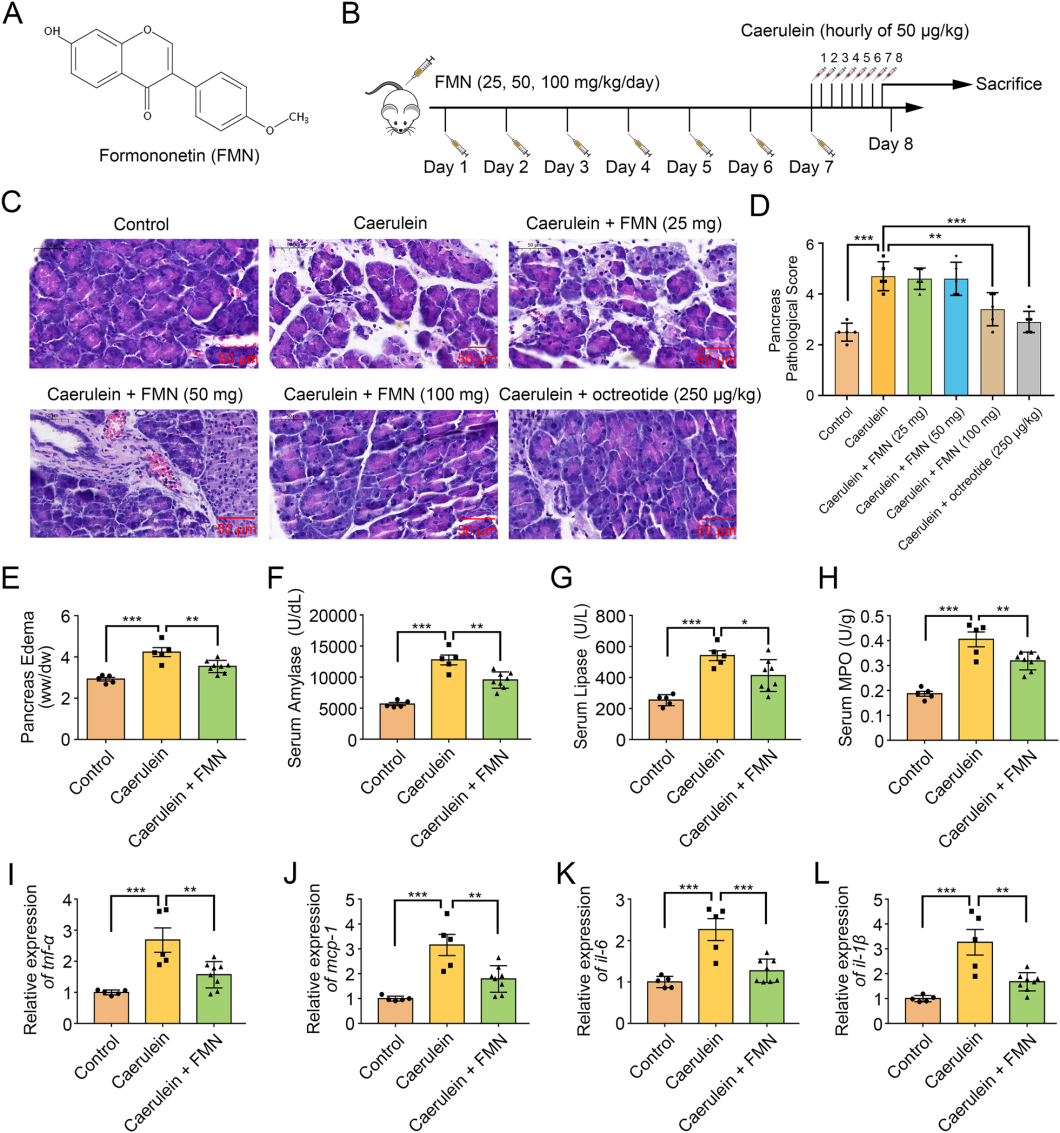
Formononetin protects against experimental AP in mice. ** A** Structure of Formononetin. **B** Schedule of experimental AP establishment. **C**, **D** HE staining of the pancreas (**C**) and histologic score (**D**); scale bar, 50 μm. **E** Detection of pancreatic edema. **F** Detection of serum amylase level. **G** Detection of serum lipase levels. **H** Detection of pancreatic MPO level. **I**-**L** qRT-PCR for pancreatic mRNA quantitative analysis of *tnf-α* (**I**), *mcp-1*(**J**), *il-6* (**K**) and *il-1β* (**L**). Octreotide was used as a positive drug. *FMN* Formononetin. Data were expressed as mean ± SD, n = 5–8 in each group. * *p* < 0.05, ** *p* < 0.01, *** *p* < 0.001

Firstly, we detected the effect of formononetin on caerulein-induced experimental AP. The chemical structure of formononetin was shown in Fig. 1A and the schedule of AP model establishment was shown in Fig. 1B. Microscopic morphological examination showed that treatment with formononetin (100 mg/kg) effectively alleviated inflammation, and pancreatic edema (Fig. 1C–E) in pancreas of AP mice. Meanwhile, the levels of serum amylase, lipase, and MPO in caerulein group were significantly increased compared to that of control, while formononetin treatment significantly decreased serum amylase, lipase levels, as well as MPO in mice with AP (Fig. 1F–H). Additionally, formononetin treatment substantially reduced pancreatic cytokine production including TNF-α, MCP-1, IL-6 and IL-1β (Fig. 1I−L). All the results above demonstrated that formononetin alleviates the severity of AP.

### Formononetin protected the integrity of the intestinal barrier and reduced bacterial translocation in AP

**Fig. 2:**
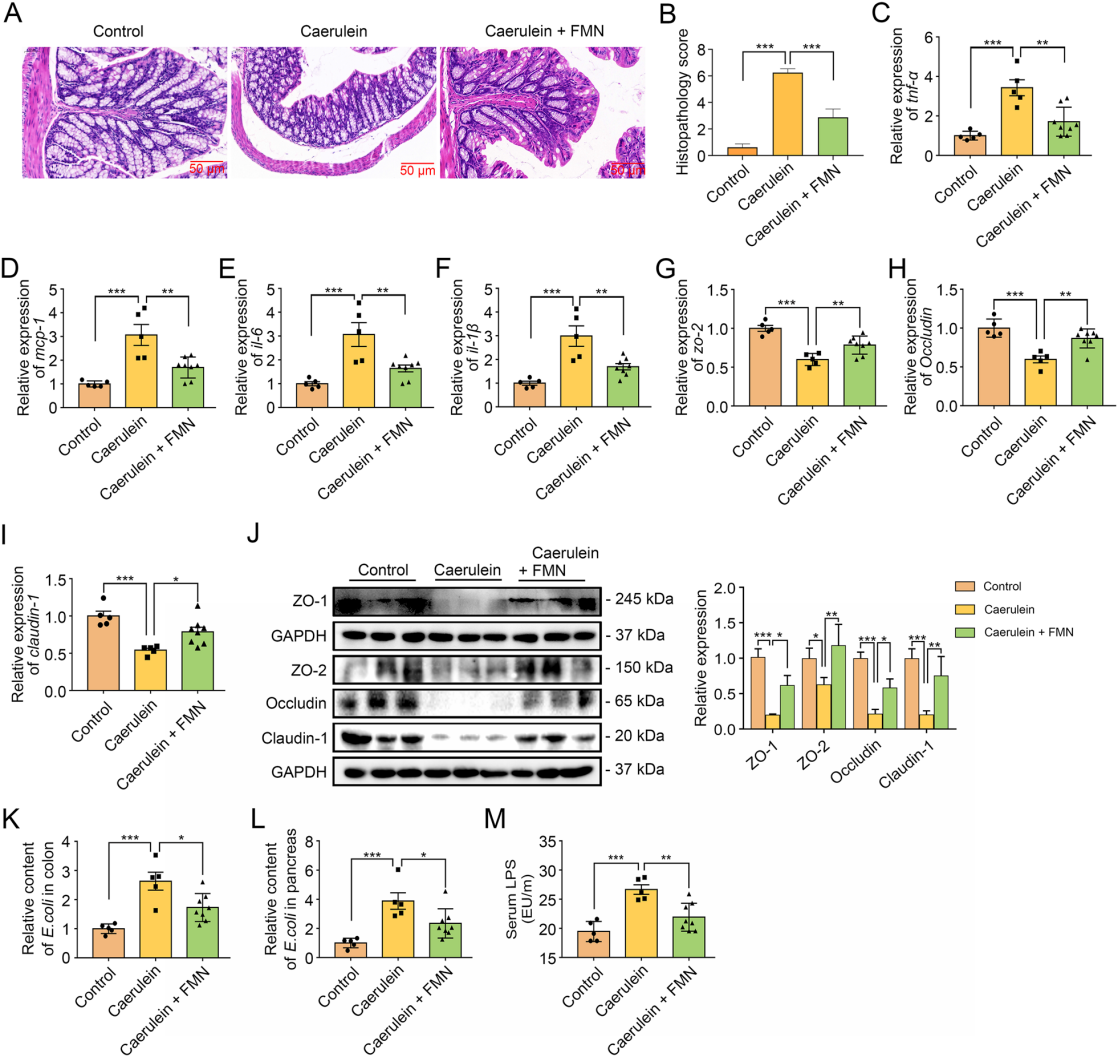
Formononetin protected the integrity of the intestinal barrier and reduced bacterial translocation in AP. C57BL/6J mice were pretreated with formononetin for 1 week and underwent induction of AP. **A**, **B** Representative H&E staining of the colon (**A**) and histopathology score (**B**); scale bar, 50 μm. **C**-**F** qRT-PCR for colonic mRNA quantitative analysis of *tnf-α* (**C**), *mcp-1* (**D**), *il-6* (**E**) and *il-1β* (**F**). **G**-**I** qRT-PCR for colonic mRNA quantitative analysis of *zo-2* (**G**), *occludin* (**H**) and *claudin-1*(**I**). Western blot and quantitative analysis of colonic ZO-2, Occludin and Claudin-1 (**J**). **K**, **L** qRT-PCR for mRNA relative content of *E. coli* in colon and pancreas. **M** Measurement of serum endotoxin. Data were expressed as mean ± SD, n = 3–8 in each group. * *p* < 0.05, ** *p* < 0.01, *** *p* < 0.001

Considering the critical role of intestinal barrier dysfunction in the development of pancreatitis [18], we next studied the effects of formononetin on intestinal injury. As showed in Fig. 2A, B, mice with AP had obvious inflammatory infiltration in colonic crypt and mucosal layer, and shortened intestinal villi, while in the group treated with formononetin, colonic damage was mitigated. By further detection of the inflammatory cytokine expression, we observed lower levels of colonic inflammation in formononetin-treated group compared to those of AP group, which was reflected by the reduced mRNA levels of inflammatory factors including *tnf-α*, *mcp-1*, *il-6* and *il-1β* (Fig. 2C-F). These results together suggest that formononetin has an anti-inflammatory effect not only in the pancreas, but also in AP-related intestinal injury. We next investigated intestinal barrier function in AP. Induction of AP caused decrease in *ZO-2*, *Occludin* and *Claudin-1* at mRNA level (Fig. 2G-I) and ZO-1, ZO-2, Occludin and Claudin-1 protein (Fig. 2J) level, which was significantly reduced by treatment with formononetin. With increased intestinal permeability, intestinal bacteria represented by *Escherichia coli* (*E. coli*) translocated through the intestinal mucosal barrier and caused pancreatic necrosis and secondary septic infection [19]. Subsequently, we detected the RNA content of *E. coli* in colonic and pancreatic tissues of AP mice, and the results showed that formononetin treatment downregulated the expression of *E. coli* mRNA induced by caerulein (Fig. 2K, L). Moreover, induction of AP caused elevation of serum endotoxin levels in mice of AP group, which was effectively mitigated by formononetin (Fig. 2M). All these results demonstrated that formononetin attenuates AP *via* enhancing intestinal barrier function and preventing bacterial translocation.

### Formononetin inhibits the activation of NLRP3 inflammasome in colon and pancreas

**Fig. 3:**
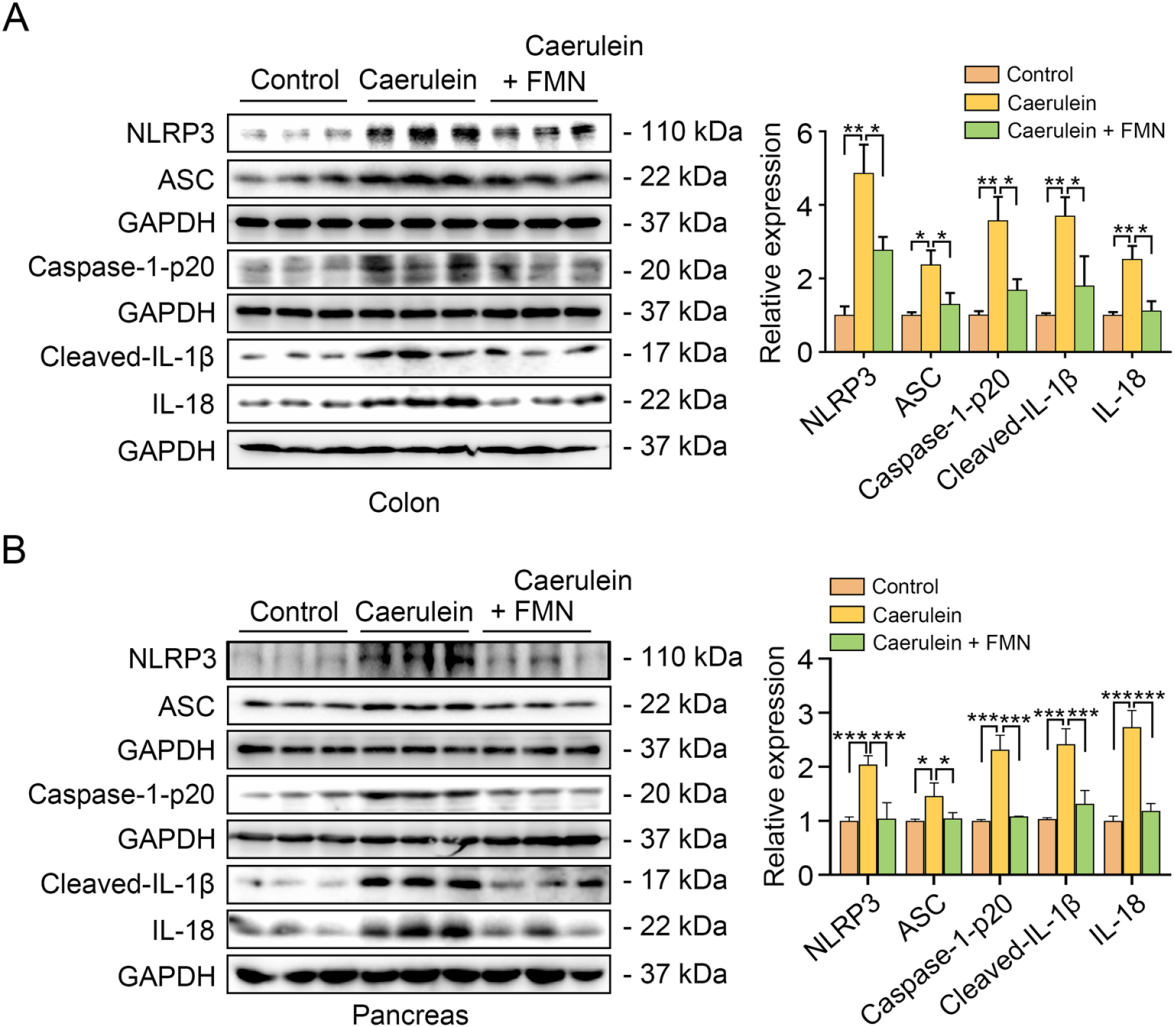
Formononetin inhibits the activation of NLRP3 inflammasome in colon and pancreas. C57BL/6J mice were pretreated with formononetin for 1 week and underwent induction of AP. **A**, **B** Western blot detection of NLRP3 inflammasome activation in colon (**A**) and in pancreas (**B**). Data were expressed as mean ± SD, n = 3 in each group. * *p* < 0.05, ** *p* < 0.01, *** *p* < 0.001

NLRP3 inflammasome pathway played an important role in AP [20]. Hence, we validated whether formononetin is implicated in modulating the activation of NLRP3 inflammasome during AP. As shown in Fig. 3A, the abundance of NLRP3, ASC, caspase-1-p20, cleaved-IL-1β and cleaved-IL-18 in colonic tissue were increased in AP mice while formononetin treatment drastically reduced the activation of NLRP3 signaling pathway. Similar to those in the colon, formononetin treatment suppressed activation of NLRP3 inflammasome in pancreas (Fig. 3B). All these data above suggested that formononetin reduced AP-induced intestinal inflammation by inhibiting the activation of NLRP3 inflammatory pathways.

### Formononetin mitigates colonic oxidative stress damage by modulating the Nrf2/Keap1 pathway

**Fig. 4:**
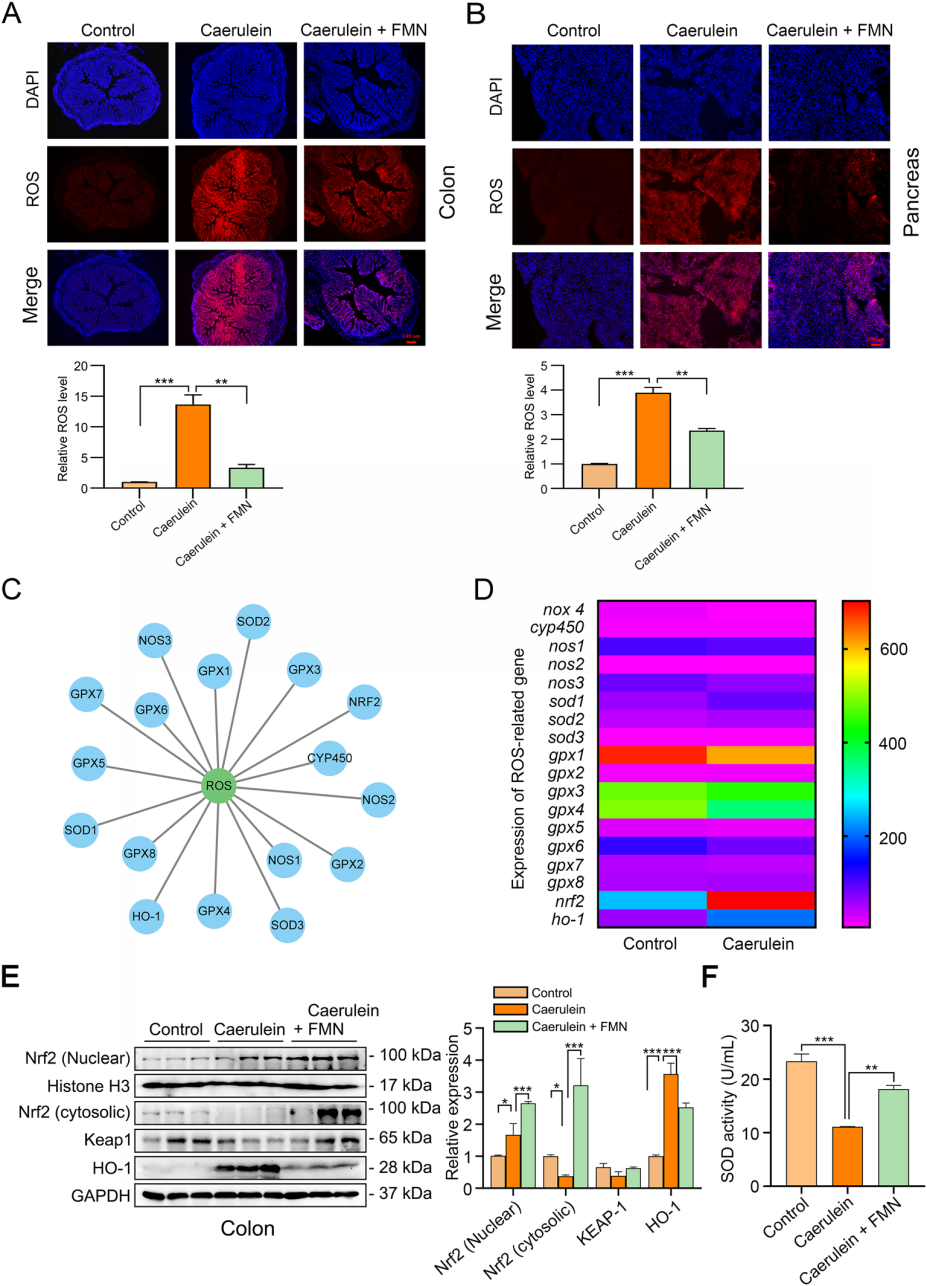
Formononetin mitigates colonic oxidative stress damage by modulating the Nrf2/Keap1 pathway. ** A** ROS levels in colon of C57BL/6J mice were detected by DHE assay. **B** ROS levels in the pancreas of C57BL/6J mice were detected by DHE assay. **C** Network analysis and visualization of ROS-related gene using cytoscape platform. **D** Analysis of ROS-related gene expression in pancreas of caerulein-induced AP from GEO database. **E** Western blot detection of Keap1/Nrf2 signaling activation in colon. **F** Detection of SOD activity in colon. Data were expressed as mean ± SD, n = 3 per group. ** *p* < 0.01, *** *p* < 0.001

As a regulator in inflammatory responses, ROS play an important role in AP [21, 22]. Therefore, we examined ROS levels in AP. Our data clearly showed that formononetin at a dose concentration of 100 mg/kg inhibits ROS levels (Fig. 4A, B). To further explore the anti-oxidative mechanisms of formononetin in AP, we detected upstream regulator of ROS. The expression of ROS-related gene in pancreas of caerulein-induced AP was obtained from GEO database (Series_geo_accession: GSE3644). As showed in Fig. 4C, D, Nrf2 showed the most significant increase after caerulein treatment. Accordingly, we detected whether there exists a change of the Keap1/Nrf2 signaling in colon. As showed in Fig. 4E, Nrf2 translocated into nuclear after caerulein treatment, interestingly, formononetin treatment significantly enhanced the abundance of Nrf2 both in nuclear and cytoplasm. There showed no significant changes in Keap1. And the Nrf2 downstream target HO-1 was elevated in AP group, while formononetin pretreatment inhibit it (Fig. 4E). SOD, another downstream of Nrf2 and a key enzyme for ROS clearance, was significantly decreased by caerulein treatment and formononetin reversed AP-induced downregulation (Fig. 4F). All these results suggest that formononetin improves oxidative stress by modulating the Nrf2/Keap1 pathway in AP-induced mice.

### Formononetin interacts with Keap1 to regulating Nrf2 signaling pathway

**Fig. 5:**
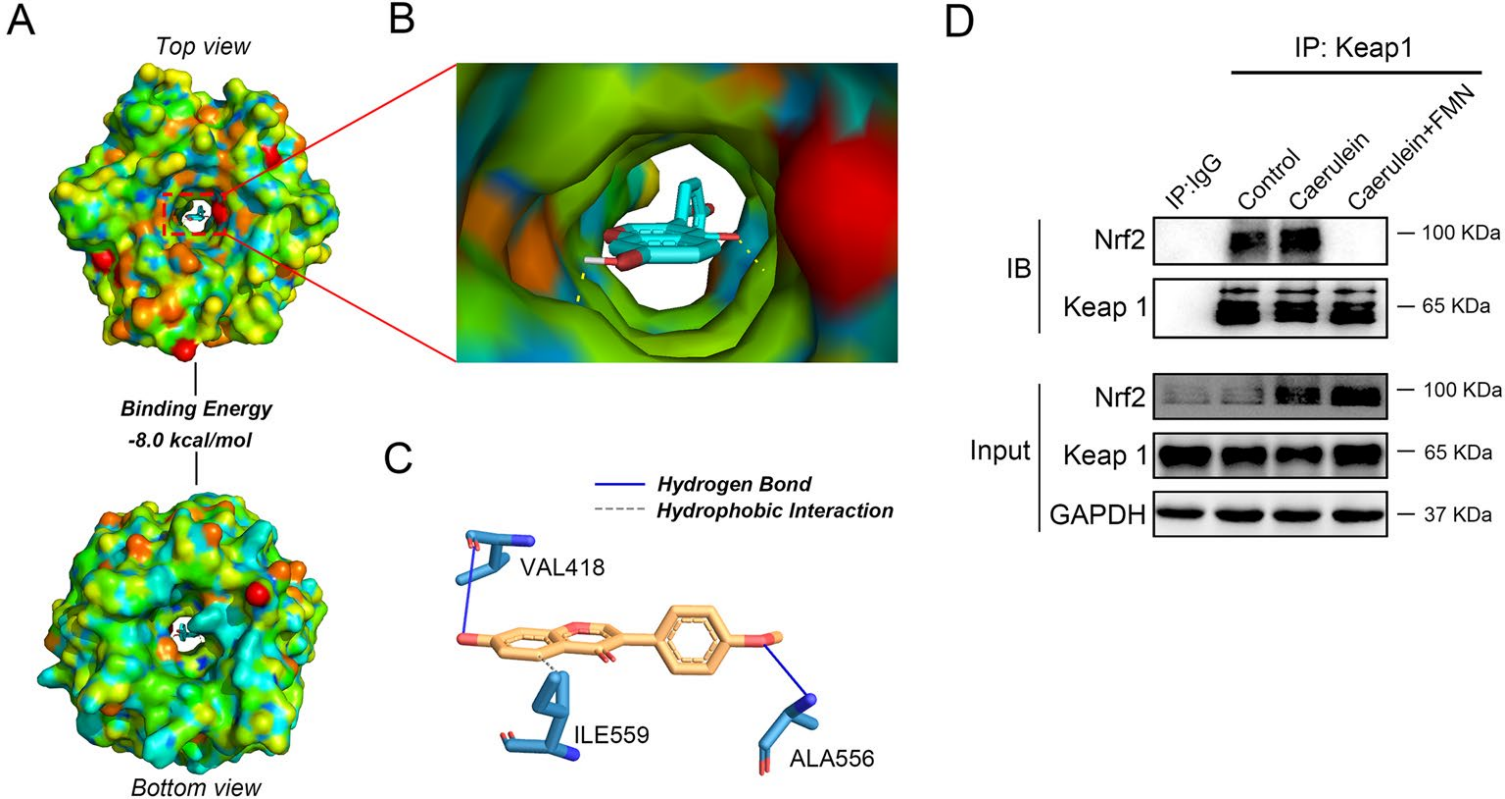
Molecular docking analysis of formononetin and the keap1 protein. Formononetin bound to the keap1 (PDB ID: 5CGJ) in the most stable pose and binding motifs was depicted with several high affinity interactions between formononetin and the keap1 pocket. Formononetin is shown as green sticks in the keap1 pocket **A**, **B**. Bonds are shown as dashed lines color-coded as follows: hydrogen bonds in blue, and hydrophobic interactions in gray **C**. *ALA* alanine, *VAL* valine, *ILE* isoleucine. **D** Co-immunoprecipitation analysis of the interaction between Keap1 and Nrf2 in Colonic tissues

Keap1 represses Nrf2 activity under quiescent conditions, whereas Nrf2 is liberated from Keap1-mediated repression on exposure to stresses [23]. Based on the result that keap1 showed no significant change in Nrf2 activation, we investigated whether formononetin interact with keap1 to modulate Nrf2. As showed in the molecular docking analysis results, the binding energy of formononetin and Keap1 was − 8.0 kcal/mol (Fig. 5A, B), which was less than the critical value of −  5.0 kcal/mol for interaction [24]. Meanwhile, identification of non-covalent interactions between formononetin and Keap1 showed that there were hydrogen bonds and hydrophobic interactions between formononetin and Keap1 (Fig. 5C). Furthermore, the Co-IP results showed that there were interactions between Keap1 and Nrf2 under caerulein treatment, while formononetin treatment block the interaction without decrease Keap1 and Nrf2 protein levels (Fig. 5D). All these results suggest that formononetin interacts with Keap1 to regulating Nrf2 signaling pathway.

## Discussion

**Fig. 6:**
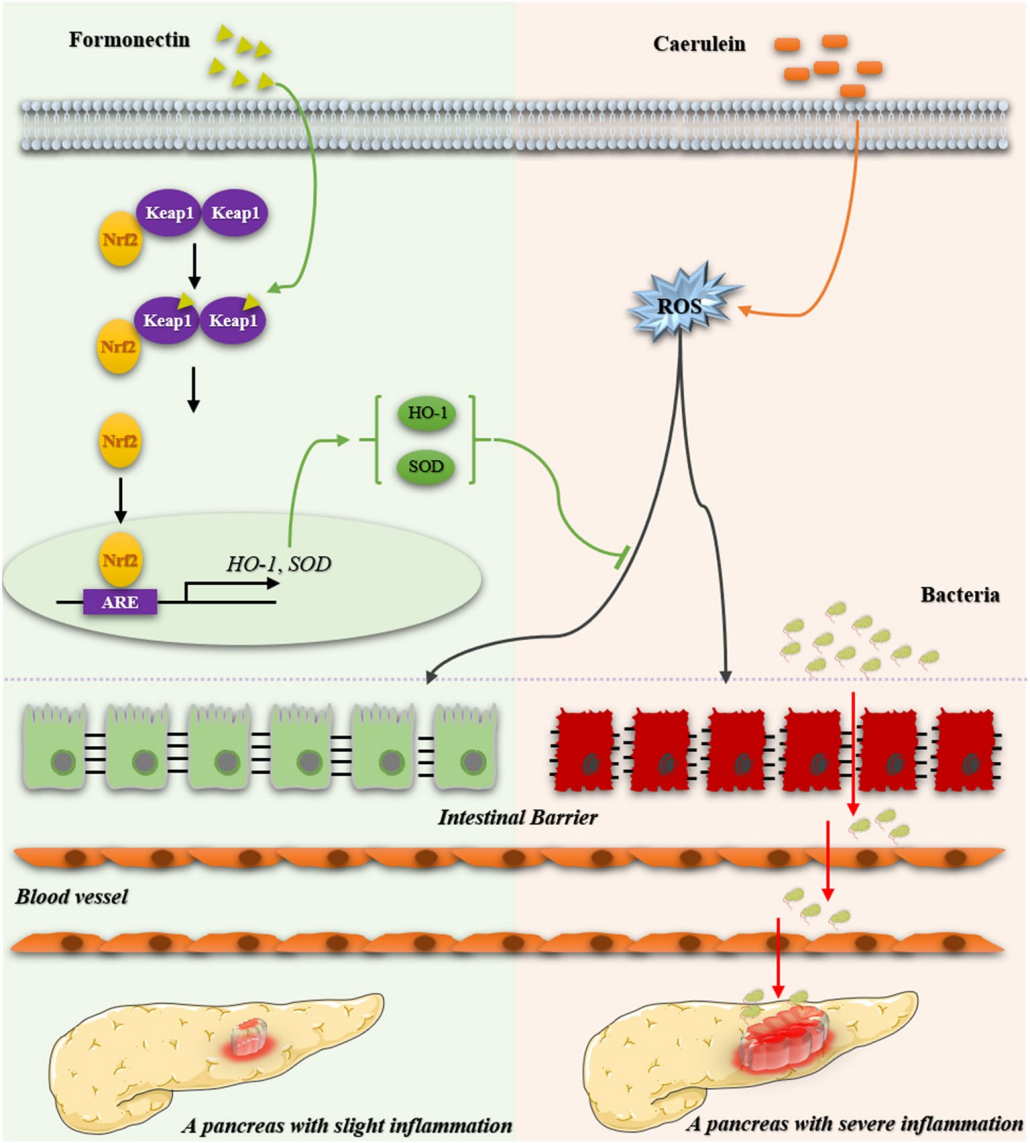
Schematic diagram of formononetin alleviates acute pancreatitis by reducing oxidative stress and modulating intestinal homeostasis

In the pathogenesis of acute pancreatitis, pancreatic damage and intestinal injury are interrelated and mutually influenced. The current study demonstrated the protective effects of formononetin on the development of experimental AP. Mechanistically, formononetin reduces ROS production by regulating Keap1 / Nrf2 signaling pathway, which further reduces ROS-mediated inflammation response, and ultimately inhibits intestinal barrier dysfunction and bacterial translocation to alleviate AP (Fig. 6).

In AP, damaged pancreatic acinar cells release inflammatory factors such as TNF-α, IL-6, and IL-1β. These inflammatory factors recruit numerous immune cells and infiltrate into the pancreatic tissue, producing more inflammatory cytokines and exacerbating the development of inflammation [25, 26]. Consistent with these, our data showed that the expression of TNF-α, MCP-1, IL-6, IL-1β, and MPO activity was significantly increased in pancreas and colon of the model group, while formononetin treatment downregulated their levels, indicating formononetin ameliorated AP and associated colonic inflammation.

ROS is closely related to the activation of the inflammatory cascade and tissue damage in acute pancreatitis. It has been recognized as an indispensable threat in the gastrointestinal tract when it comes to host defense and redox signaling [27]. When ROS is over-produced, oxidative stress will occur in cells, leading to damaging consequences, including DNA damage, lipid peroxidation, and protein modification. [28]. These various biological cascades ultimately cause cell death [29]. It’s worth noting that the intestine is more vulnerable to excess ROS than other organs due to continuous exposure [30]. By detecting ROS production in the pancreas and intestine of AP mice, we demonstrated that AP stimulation induced ROS production in pancreas and more ROS production in intestinal epithelium, and formononetin significantly inhibits ROS accumulation, thereby improving the oxidant/antioxidant balance. In addition, accumulating evidence confirms that Keap1/ Nrf2-ARE is an important antioxidant signal for ROS clearance and has a protective effect on AP [31]. Consistently, our study demonstrated that the expression of Keap1/ Nrf2-ARE was significantly down-regulated in AP mice, and their downregulation can be reversed by formononetin administration, which further confirms the anti-oxidative function of formononetin in the context of AP. Along with Nrf2 activation, the downstream antioxidative enzymes HO-1 or SOD will be activated to degrade ROS [32, 33]. By further analysis of these two enzymes protein expression or activity, we deciphered that formononetin exerts antioxidant effect during AP through up-regulation of SOD but not HO-1.

NLRP3 is an important receptor for pathogen recognition, such as substances released by cell damage, bacteria as well as bacterial toxins. Once recognized by the upstream sensor, the apoptosis-associated speckle-like protein ASC acts as a bridge connecting the sensor to the downstream effector cysteine protease caspase-1 [34]. Activated caspase-1 cleaves the precursor cytokines pro-IL-1β and pro-IL-18 to produce their bioactive forms (IL-1β and IL-18, respectively) [35]. It has been reported that deletion of caspase-1, ASC or NLRP3 significantly reduced edema and inflammation of AP [36]; previous studies also indicated that knockdown of caspase-1 significantly reduced the degree of death and inflammation of pancreatitis follicle cells [37]. Another study using NLRP3-deficient mice or the NLRP3 inhibitor INF-39 discovered that maturation and release of IL-1β were inhibited and further prevented the inflammatory cascade in a caerulein + LPS-induced AP model [38]. We found that in colonic tissue, formononetin pretreatment group significantly reduced the high expression of NLRP3, ASC, caspase-1-p20, cleaved-IL-1β, and cleaved-IL-18 induced by AP, suggesting that formononetin may reduce inflammation in the colon by inhibiting the activation of NLRP3 inflammatory vesicles. This is consistent with the previous finding that formononetin administration attenuates colitis by inhibiting the NLRP3 inflammasome signaling pathway [13]. Similarly, results from pancreas also suggested that formononetin alleviated inflammation by inhibiting the activation of NLRP3 inflammasome signaling pathway.

In addition, prior studies have demonstrated that translocation of intestinal bacteria exacerbate AP through TLR4 mediated activation of NF-κB, and thereby up-regulating the mRNA and protein expressions of NLRP3, Pro-IL-1β and Pro-IL-18 [39]. In our study, we consistently demonstrated higher *E. coli* expression in pancreas of AP mice than the control group, indicating that intestinal bacterial translocation is likely to trigger the activation of NLRP3 related pathway proteins. As leaky gut contributes to the transfer of bacteria and toxins to the pancreas in acute pancreatitis [40], we thereafter evaluated the intestinal barrier function to decipher the protective mechanism of formononetin on AP and associated intestinal injury. Interestingly, we demonstrated that formononetin treatment enhanced intestinal barrier integrity by upregulating tight junction proteins. Thus, formononetin inhibited the reproduction of *E. coli* in the intestine and its subsequent transfer to the pancreas during AP, thereby preventing secondary infection and inflammatory cascade in pancreas [36, 41, 42].

## Conclusion

In this study, we found that formononetin can reduce the severity of pancreatic injury, maintain intestinal barrier homeostasis, and alleviate inflammation and oxidative damage in AP. The protective mechanism may be related to the regulation of Nrf2/Keap1 cascade and ROS-mediated NLRP3 pathway activation. Our study provides a new experimental basis for the clinical treatment of AP.

## Supplementary Information

**Additional file 1**: **Table S1**.Specific primer sequencesused for each gene.

**Additional file 2**: **Figure S1**.Effect offormononetin on serum amylaseand lipase activity. (A) Detection of serum amylase level. (B) Detection of serum lipase levels.Data were expressed as mean ± SD, n = 6 per group. * *p* < 0.05, ** *p* <0.01, *** *p* < 0.001.

## Data Availability

All data and related information were included in this published paper, which could be acquired in the figures, tables and supplemental materials. Data of this study is available by the authors, without undue reservation.

## Abbreviations

AP: Acute pancreatitis
ASC: Apoptosis-associated speck-like protein containing CARD
DAPI: 4′, 6-diamidino-2-phenylindole, dihydrochloride
DMSO: Dimethyl sulfoxide
*E. coli*: *Escherichia coli*
HO-1: Heme oxygenase-1
IL-1β: Interleukin-1 beta
Keap1: Kelch Like ECH associated protein 1
LPS: Lipopolysaccharide
MAPK: Mitogen-activated protein kinase
MPO: Myeloperoxidase
NLRP3: Nucleotide-binding oligomerization domain leucine-rich repeat and pyrin domain-containing 3
Nrf2: Nuclear factor erythroid2-related factor 2
OCT: Optimal cutting temperature
PBS: Phosphate buffer saline
qRT-PCR: Quantitative real-time PCR
ROS: Reactive oxygen species
SDS-PAGE: Sodium dodecyl sulfate polyacrylamide gel electrophoresis
SOD: Superoxide dismutase
TJPs: Tight junction proteins
TNF-α: Tumor necrosis factorα
ZO-2: Zonula occludens-2
