# Supplementary material for: Formononetin alleviates acute pancreatitis by reducing oxidative stress and modulating intestinal barrier
Source: Chin Med. 2023 Jun 27;18:78. doi: 10.1186/s13020-023-00773-1 (PMC10304236; doi:10.1186/s13020-023-00773-1)
Supplement: Supplementary file 1 — Additional file 1: Table S1.Specific primer sequencesused for each gene. [file 13020_2023_773_MOESM1_ESM.docx]

**[Table 1](https://www.frontiersin.org/articles/10.3389/fmicb.2021.665184/full#SM1)**. Specific primer sequences used for each gene

| Gene | Forward（5’-3’） | Reverse（5’-3’） |
| --- | --- | --- |
| TNF-α | CCCTCACACTCAGATCATCTTCT | GCTACGACGTGGGCTACAG |
| Interleukin-6 | TAGTCCTTCCTACCCCAATTTCC | TTGGTCCTTAGCCACTCCTTC |
| IL-1β | GCAACTGTTCCTGAACTCAACT | ATCTTTTGGGGTCCGTCAACT |
| MCP-1 | TTAAAAACCTGGATCGGAACCAA | GCATTAGCTTCAGATTTACGGGT |
| ZO-2 | ATGGGAGCAGTACACCGTGA | TGACCACCCTGTCATTTTCTTG |
| Occludin  Claudin-1  *Escherichia coli* | TTGAAAGTCCACCTCCTTACAGA  GGGGACAACATCGTGACCG  CATGCCGCGTGTATGAAGAA | CCGGATAAAAAGAGTACGCTGG  AGGAGTCGAAGACTTTGCAC  T  CGGGTAACGTCAATGAGCAAA |
| β-Actin | CCCAGGCATTGCTGACAGG | TGGAAGGTGGACAGTGAGGC |
