# Supplementary material for: Formononetin alleviates acute pancreatitis by reducing oxidative stress and modulating intestinal barrier
Source: Chin Med. 2023 Jun 27;18:78. doi: 10.1186/s13020-023-00773-1 (PMC10304236; doi:10.1186/s13020-023-00773-1)
Supplement: Supplementary file 2 — Additional file 2: Figure S1.Effect offormononetin on serum amylaseand lipase activity. (A) Detection of serum amylase level. (B) Detection of serum lipase levels.Data were expressed as mean ± SD, n = 6 per group. * p < 0.05, ** p <0.01, *** p < 0.001. [file 13020_2023_773_MOESM2_ESM.docx]

**Figure S1**


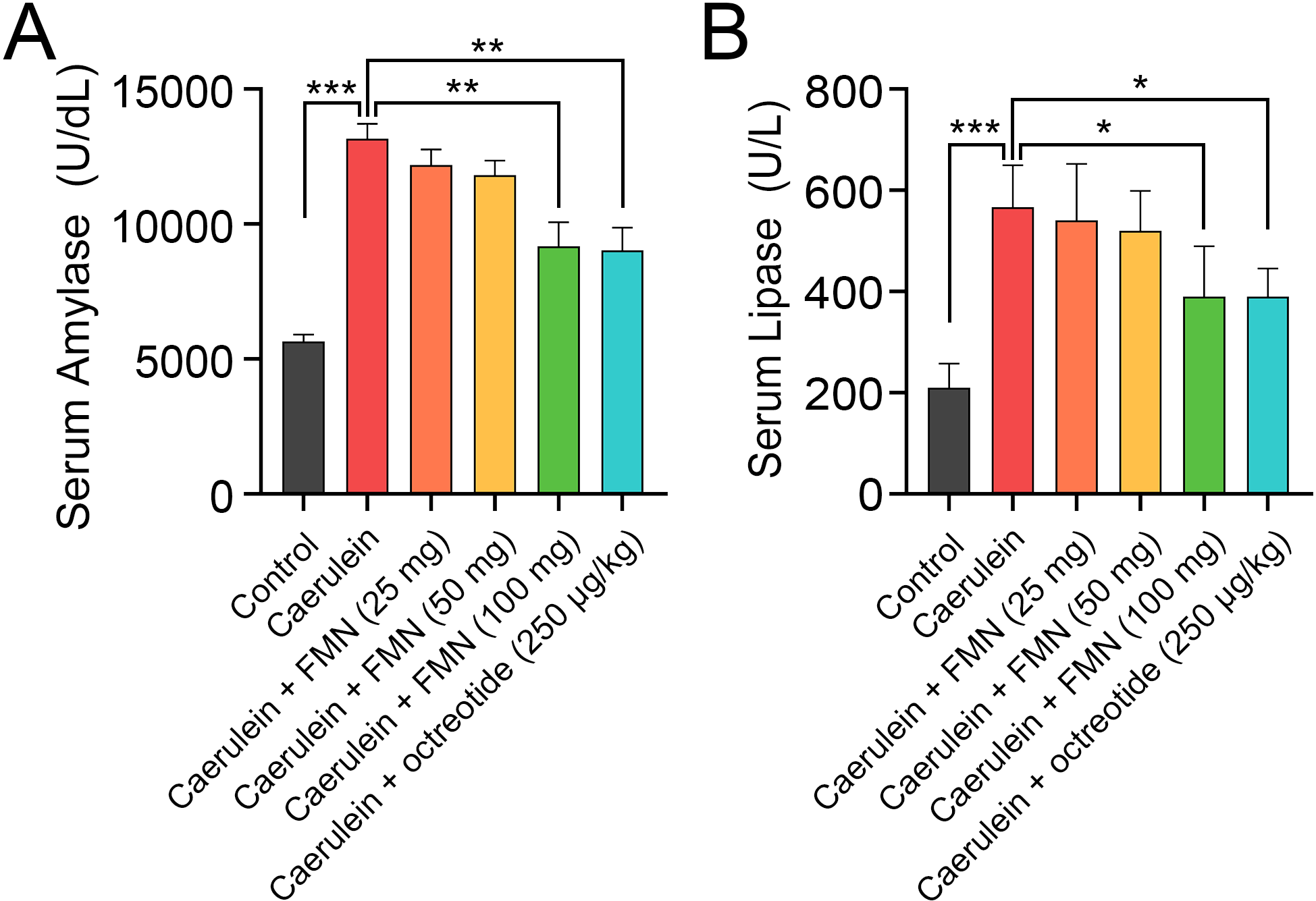


**Fig. S1**. Effect of formononetin on serum amylase and lipase activity. (A) Detection of serum amylase level. (B) Detection of serum lipase levels. Data were expressed as mean ± SD, n = 6 per group. * *p* < 0.05, ** *p* < 0.01, *** *p* < 0.001.
